# Structures, Properties and Applications of Alginates

**DOI:** 10.3390/md20060364

**Published:** 2022-05-29

**Authors:** Roya Abka-khajouei, Latifa Tounsi, Nasim Shahabi, Anil Kumar Patel, Slim Abdelkafi, Philippe Michaud

**Affiliations:** 1 Department of Food Science and Technology, College of Agriculture, Isfahan University of Technology, Isfahan 84154, Iran; r_abka@yahoo.com; 2 Institut Pascal, Université Clermont Auvergne, CNRS, Clermont Auvergne INP, F-63000 Clermont-Ferrand, France; latifa.tounsi@enis.tn; 3 Laboratoire de Génie Enzymatique et Microbiologie, Équipe de Biotechnologie des Algues, Département Génie Biologique, Ecole Nationale d’Ingénieurs de Sfax, Université de Sfax, Sfax 3038, Tunisia; slim.abdelkafi@enis.tn; 4 Department of Food Hygiene and Quality, College of Veterinary Medicine, Shahrekord 88186, Chahar Mahal Bakhtiari, Iran; nasim_shahabi88@yahoo.com; 5 Department of Marine Environmental Engineering, National Kaohsiung University of Science and Technology, Kaohsiung City 81157, Taiwan; anilkpatel22@gmail.com

**Keywords:** alginate, polysaccharide, seaweeds, hydrocolloids

## Abstract

Alginate is a hydrocolloid from algae, specifically brown algae, which is a group that includes many of the seaweeds, like kelps and an extracellular polymer of some bacteria. Sodium alginate is one of the best-known members of the hydrogel group. The hydrogel is a water-swollen and cross-linked polymeric network produced by the simple reaction of one or more monomers. It has a linear (unbranched) structure based on d-mannuronic and l-guluronic acids. The placement of these monomers depending on the source of its production is alternating, sequential and random. The same arrangement of monomers can affect the physical and chemical properties of this polysaccharide. This polyuronide has a wide range of applications in various industries including the food industry, medicine, tissue engineering, wastewater treatment, the pharmaceutical industry and fuel. It is generally recognized as safe when used in accordance with good manufacturing or feeding practice. This review discusses its application in addition to its structural, physical, and chemical properties.

## 1. Introduction

Sodium alginate was first discovered in Kelp in 1883 and has been extensively studied by many researchers since this date [1]. Alginates are anionic hydrophilic heteropolysaccharides that are abundant in nature that exist both as components in brown seaweed (Phaeophyceae) and as capsular polysaccharides of some soil bacteria [2]. Macroalgae are a source of many hydrocolloids. Based on their pigment content they are divided into brown, green and red algae [3].Each of them has a specific and predominant matricial polysaccharide, and that of brown seaweeds is alginate. Although there are several species of brown seaweed containing alginate, the majority of them are not abundant enough and are not in a good place for commercial production [4]. These algae contain pigments composed of the chlorophylls a and c, which are covered by carotenes and xanthophylls. Phycoxanthin (xanthophyll) is responsible for their brown color.

Polysaccharides extracted from brown algae are alginates, fucoidans (a backbone of *α*(1→3)-l-fucopyranose residues or of alternating *α*(1,3) and *α*(1,4)-linked l-fucopyranosyls periodically interrupted by other monosaccharides and sulfate ester groups at two, three and/or four positions of fucopyranose units) and laminaran (a storage β-(1,3) glucan occasionally containing β-(1,6)-linked branches). Fucoidan and laminaran are mostly used for their biological activities, while alginates have many applications in the food and medical industries such as thickeners, emulsifiers, stabilizers, and pharmaceutical additives [3,5,6,7].

Alginates consist of (1,4) linked β-d-mannuronic and α-l-guluronic acids, both of them in pyranosic conformation, arranged in homogeneous (MM or GG) and heterogeneous (MG or GM) blocks [3,8], leading to a large diversity of structures, molecular weights, and physicochemical properties. Alginate production by bacteria was first reported in the opportunistic pathogen *Pseudomonas aeruginosa* and then in three non-pathogenic species of *Pseudomonas* including *P. mendocina, P. putida* and *P. fluorescens.* Soil bacteria (*Azotobacter vinelandii*) is very suitable for the production of bacterial alginates [9].

**Figure 1:**
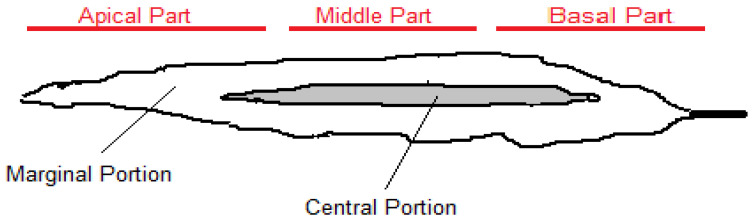
Different parts of a blade of *Saccharina japonica*.

Commercial alginates are produced using *Laminaria hyperborea*, *Laminaria digitata*, *Macrocystis pyrifera*, *Ascophyllum nodosum*, *Ecklonia maxima*, *Saccharina japonica* (formerly *Laminaria japonica*), *Lessonia nigrescens*, *Durvillea antarctica* and *Sargassum* spp [10]. It is estimated that 23,000 tons of alginate are produced from about 85,000 tons of algae annually [11]. Commercial brown seaweed such as *Laminaria Ecklonia* and *Macrocystis* and to some extent *Sargassum* contain valuable sodium alginate. Depending on the source, this polyuronide is estimated to be up to 40% of their dry weights [12]. The physical and chemical properties of alginates depend on how each monomer is placed in the chain, and their molecular weight [10,13]. In addition to the aforementioned features, the length of the uronic acid chain and the percentage of each monomer (meaning guluronic and mannuronic acids) are also important. These factors cause significant structural differences as well as specific physico-chemical properties [11]. The abundance, composition and M/G ratio of alginate can vary not only by plant species and algae age but also by natural alginate source, plant location, geographical location, and season. These factors affect the functional properties of alginate, solubility, reaction with metal ions, viscosity, and gel-forming properties [10,11]. Some reports on the structure of alginates from *Sargassum* and *Turbinaria* indicate that they have a low M/G ratio. Alginates from these species found in hot water can be very useful in cases that require the formation of strong gels. Moreover, alginates from species found in cold water often have poor viscosity [14,15,16]. The monomeric ratios (M/G) of sodium alginate vary in different species of brown algae. For example, alginate from *Sargassum* has an M/G ratio of 0.8 to 1.5, compared to 2.26 in *Laminaria* [10,11,12,13]. In addition, the M/G ratio of sodium alginate from *Sargassum miyabei* has been decreasing from March to August because the amount of G monomer has increased over time. A study conducted during spring, summer and autumn found that the amount of alginate obtained from *Sargassum filipendula* varies from 15.1% to 17.2% based on dry weight. Spring algae were the richest source for the extraction of this polymer and the ratio of M/G in all samples of these algae is < 1 (Table 1) [11]. Therefore, extracted alginate with this property is suitable for obtaining food resistance gels and industrial applications [11]. In another study, different parts of *Saccharina japonica* were compared in terms of monomer ratios, and it was found that the M/G ratio is as follows: basal- > central-> apical parts and the central portion > marginal portion > apical part (Figure 1). This probably means that the base part (including the growth point) of *S. japonica* always synthesizes M, which gradually turns into G with aging and elongation of the blade by epimerization at the polysaccharide level [17].

**Figure 2:**
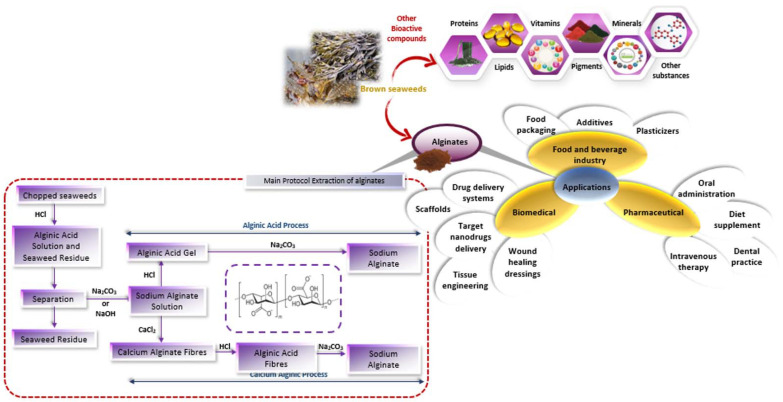
Production process and applications of alginates.

The extraction of alginate can be summarized in five steps (Figure 2). First, the dried and crushed brown seaweeds were extracted with a mineral acid (e.g., HCl, 0.1 M), leading to insoluble alginic acids which are easily separated from other contaminating glycans such as sulfated fucoidans and laminarans by filtration or centrifugation. The insoluble residue is then treated by alkaline solution (using sodium carbonate, sodium hydroxide or aluminum hydroxide, above pH = 6.0) to convert insoluble alginic acid into sodium alginate. After another separation step, the soluble sodium alginate is precipitated using calcium chloride or cold alcohol. Alginates are then purified using techniques such as acidification, the addition of Ca ^+2^ ions (calcium alginate formation), or the addition of ethanol (dielectric stabilizer) [10].

## 2. Molecular Structure of Alginate

**Figure 3:**
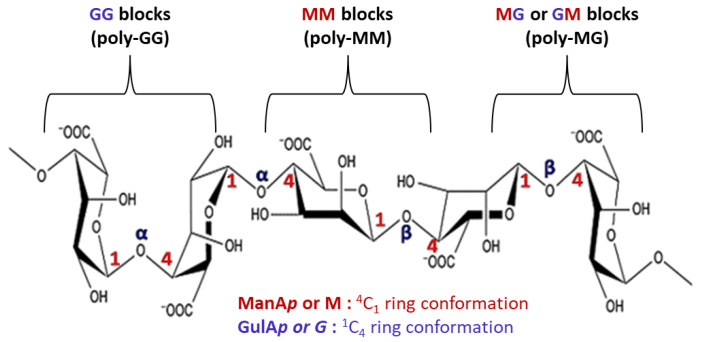
Chemical structure of alginate.

Alginates are natural anionic polysaccharides belonging to the family of linear copolymers (without branches) found in the cell wall matrix of brown seaweed [12]. The main structure of alginates is composed of two monomeric units: β-(1,4) linked d-mannuronic acid (ManA*p* or M) with ^4^C_1_ ring conformation and α-(1,4)-linked l-guluronic acid (GulA*p* or *G*) with ^1^C_4_ ring conformation [35] (Figure 3).

The structural characterization of alginate includes elementary biochemical assays but also more complex analyses (spectroscopy, chromatography) leading to fine structure identification. Chemical assays are expected to quantify both sugar content (total sugar, neutral sugar and uronic acids) and non-sugar contaminants co-extracted with seaweed alginates [27], such as phenolic compounds, proteins and sulfated groups. High performance anion exchange chromatography with pulsed amperometric detection (HPAEC-PAD) and sas chromatography coupled with mass spectrometry and electron ionization (GC/MS-EI) have been widely used for the determination of M/G ratios of alginates after their complete acid hydrolysis using formic acid (90% *v/v*) and trifluoroacetic acid (2 M, 90 min, 120 °C), respectively [27].

**Figure 4:**
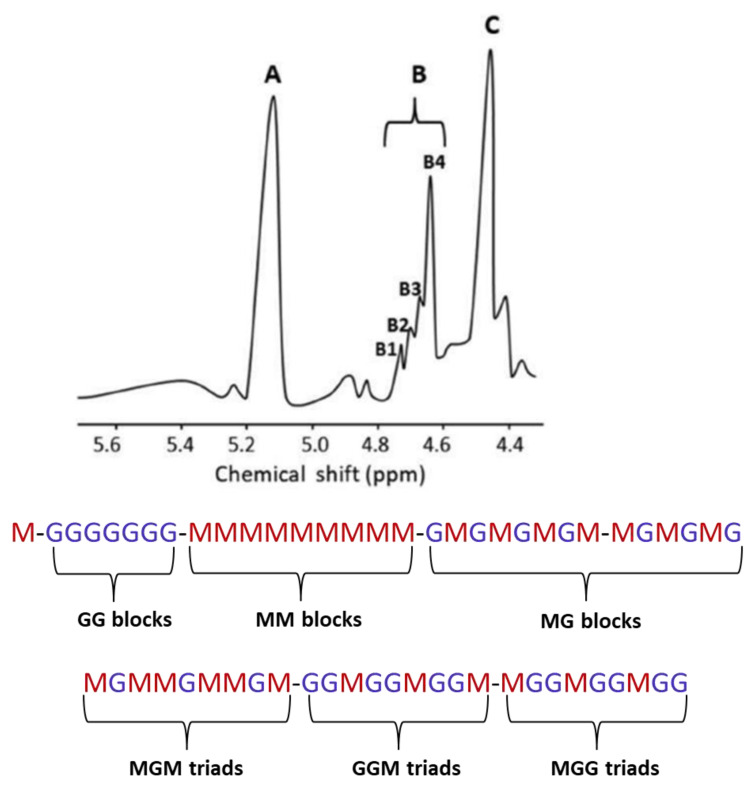
Possible sequences of l-guluronic (G) and d-mannuronic (M) acids in alginate. Signal B2 (4.70–4.75 ppm), B1 (4.74–4.78 ppm) and signal at 4.42–4.44 ppm refer to the H-5 of the central G in MGM, GGM or MGG triads, respectively. The H-1 of M neighboring M (signal B4) and G (signal B3), respectively, are presented at 4.66–4.68 (MM) and 4.68–4.70 (MG) ppm.

^1^H NMR spectroscopy is the main method used to study the composition and structure of alginate [15] (Figure 4). Five species of brown algae from the Egyptian Red Sea coasts (Sirophysalis trinodis-formly *Cystophyllum trinode*, *Polycladia myrica*-formly *Cystoseira myrica*, *Sargassum dentifolium*, *Sargassum asperifolium* and *Sargassum latifolium*) were investigateded using ^1^H NMR spectroscopy [18]. The analysis revealed a guluronic acid (G) content higher than the mannuronic (M) one and had a homopolymeric block-type structure. Normally, alginate is an insoluble salt of all the cations (the main ones being sodium, magnesium, and calcium) that are found in seawater and is rapidly ion exchanged with seawater. Therefore, alginate extraction can be considered as a process in two stages: the conversion of insoluble alginate to soluble form followed by the diffusion of the soluble glycuronan into solution [18].

Fundamentally, alginates are characterized by their molecular mass (M_w_, M_n_), polydispersity index (M_w_/M_n_), macromolecular parameter (e.g., intrinsic viscosity ([η]), critical concentration (C*), gyration (R_g_) and hydrodynamic qualities (R_h_) radii), as well as by their M/G ratio and number and length of monad (G and M), diad (GG, MM, MG or GM), and triad (MMG, GGM, MGM) frequencies, which provide structural information that is easily correlated with their rheological properties (gelling or/and thickening) in a solution or in the presence of mono- (K^+^, Na^+^) and di-valent (Ca^2+^, Mg^2+^) salts [27]. The arrangement of G and M blocks in alginates, depending on their natural source, can be sequential (repeating units of GGGG or MMMM blocks), alternating (GMGMGMGM) or random [10] (Figure 4) (Table 2). The linear and flexible structure of alginate consists of a steric barrier around the carboxyl groups where the G-blocks form folded and rigid structures [36]. Alginates are commercially available as sodium, potassium, or ammonium salts. The molecular weight of commercial alginate is usually from 60,000 to 700,000 Daltons, depending on the application [35].

Alginates contain many free hydroxyl (-OH) and carboxyl (-COOH) groups that enable them to form intramolecular hydrogen bonds [10]. Alginate oligosaccharides (AOS) are oligomers containing two to 25 monomers which can be obtained by the hydrolysis of glycosidic bonds, organic synthesis or by biosynthesis [38,39,40]. Examples of these methods are, respectively, the depolymerization of alginate by H_2_O_2_, the production of a pentamer of mannuronic acid using an orthogonal glycosylation strategy, and the production of AOS from glucose by a culture of *Pseudomonas mendocina* NK-01 under nitrogen starvation. AOS have a shorter chain length, resulting in improved solubility compared to higher molecular weight alginates. They have received more attention due to their low molecular weights and higher biological activities that are sometimes associated with health benefits. They have immunomodulatory, antimicrobial, antioxidant, probiotic, antihypertensive, anti-diabetic, anti-tumor, anticoagulant, light-protecting, antibacterial, anti-lipid, anti-hypertensive, and anti-hypoglycemic properties as well as the ability to suppress obesity and enhance cell proliferation [39,40,41]. They also regulate plant growth. For example, they have been used as prebiotics, dietary supplements for aquaculture, poultry and pigs, plant, and microbial growth stimulants, cryoprotectants for frozen foods, and post-harvest treatments [39,41,42,43].

## 3. Modification of Alginate by Processing Methods

As the properties of polysaccharides are tightly related to their structures, the modification of alginate sometimes affects its properties. The molecular weight reduction of the alginate occurs through a uronic acids release by proton catalyzed hydrolysis in acid conditions (pH < 5), and elimination reaction in neutral and alkaline conditions (pH > 5) [44,45]. Microwave-assisted acidic hydrolysis of alginate has the same effect as normal acid hydrolysis, but it accelerates the reaction [46]. Ultrasound treatments of alginate at different frequencies cause polymer structure degradation, rearrangement, and alteration of its molecular weight, reducing the M/G ratio (changing hydrophobic interactions). As a result, they are harder than untreated polymers [47,48]. High-power electrical energy (several tens of kilojoules) is used in the high-voltage electrical discharge method. If the electric field is strong enough, an electron avalanche will be the starting point for the spread of the streamer from the high-voltage needle electrode to the plate electrode. High-pressure shock waves, bubble cavitation, and fluid turbulence are produced and lead to partial decomposition and damage to the cell wall, which accelerates the extraction of biomolecules from biomass. Studies have shown that the molecular weight of alginate extracted by applying the high voltage method was similar to that extracted with the classical method but had a higher polydispersity. Alginate fragmentations and degradations occurred leading to heterogeneity in M_w_ distribution. This method had logically no effect on the sequence ratio of this biopolymer [49]. Molar mass, polydispersity and the intrinsic viscosity of alginate falls simultaneously during ultra-high-pressure homogenization (HPH) without any change in its conformational structure [50].

## 4. Physical Properties of Alginate Gel

### 4.1. Gel Formation

**Figure 5:**
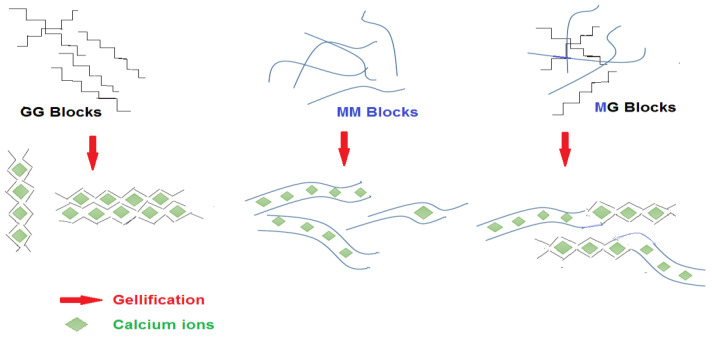
Effect of calcium on the MG, MM and GG alginate Units.

Compared to other polysaccharides such as gelatin or agar, alginate is able to form gels independently of temperature changes. The formation of alginate gels can be achieved by two methods of ionic bonding with cations (ionic gels) or acid deposition (acidic gels) [35]. Alginates in solution give pseudoplastic (shear-thinning behavior) liquids. It was also observed that at constant concentrations and temperatures, the thickening properties of several alginates from different algal sources, regardless of their origin, are only correlated to their molecular mass. The formation of alginate gels is a very complex process and depends on factors such as the type of alginate used (e.g., M/G ratio), the degree of conversion to calcium alginate, the source of calcium ions (calcium chloride, phosphate, lactate or acetate) and its preparation methods [51]. In the presence of divalent cations such as calcium, a strong interaction occurs between ions and COO^−^ groups of guluronic acids from different chains, creating a three-dimensional lattice insoluble in water and thermally irreversible. This structure is often referred to as the “egg box” [36]. Alginate gel formation due to the higher degree of barrier to rotation around glycosidic bonding depends on the percentage of G and M units. Because of this configuration, cross-linking between alginate and Ca^2+^ ions are mostly due to the presence of G units [47,52] (Figure 5).

### 4.2. Gel Power

The most useful property of alginates is their ability to react with cations, especially multivalent cations such as calcium ions, to produce strong gels or insoluble polymers [53]. The chains and the structure of the gel depends on the sequence and composition of the alginate chain, which ultimately determines its stiffness. M units are soft and elastic and cause a delay in gel formation. Elastic gels are composed of alginate with high M contents (M/G > 1), while gels are obtained from alginate with a low M/G ratio, (M/G < 1) and are compact, hard, and brittle. The order of chain stiffness is MG < MM < GG. Gels prepared from low molecular weight alginate and high G units are the strongest and best gels for encapsulation and have been used to protect probiotics [2,32,54,55]. The strength of the gel also depends on the degree of interaction of the alginate with the divalent cation and the ionic radius. For example, Ba^+2^ ions are more likely to react with alginates than Ca^+2^ ions. Ba^+2^ ions are attached to M- and G-blocks but Ca^+2^ ions are binded to G- and MG blocks. Therefore, Ba^+2^ ions produce a stronger gel than Ca^+2^ ones [56]. The gel strength of alginates varied from 10.97 to 15.51 (N/cm^2^) [18].

### 4.3. Rheology of Alginate Gels

Rheological behavior is an important parameter for the application of polysaccharides in the food industry [32]. The oscillatory rheology is generally used to quantify the viscous and elastic responses at different time scales of viscoelastic systems. The storage or elastic modulus (G’) describes the elastic properties, while the viscosity or loss or plastic modulus (G”) is proportional to the viscous resistance [57]. Alginate gel particles are soft viscoelastic particles that deform in response to external stimuli due to the presence of water in their gel network [35]. Different molecular weights and different amounts of guluronate and manuronate monomers, alginate type, concentration and gelling ion affect gel rheology. The rheology of alginate from bacteria and seaweed is different according to these properties [57]. A higher elastic modulus in the gels that were formed with higher molecular weight alginate was observed. The faster availability of shorter chains during gelling is probably the reason for the dependency of gel rheology to M_w_. Therefore, the gelling rate can be reduced by using high M_W_ alginates. Also, high-M alginate gels were more elastic than the high-G ones because G sequences form junctions with divalent cations. Particle size affects the rheology of the gel in such a way that a higher rate of gelation and a lower final storage modulus are results of using smaller particles, whereas larger particle size causes lower gelling rates and higher gel elasticity. The particles size dependency may be interpreted as the result of the change in the amount of particle surface. In this way, in smaller particles, an increase in surface area per total particle weight happens which leads to a higher rate of calcium ion release into the gel-forming mix and causes a lower gelation half time and lower elastic modulus in the final gel [58,59]. The temperature is an external factor that has an effect on the gel so that when the temperature increases, the elasticity of the gel decreases and the gel becomes more viscous [49].

### 4.4. Porosity and Permeability

Small soluble molecules, such as glucose and proteins as small as insulin are able to disperse into and out of alginate granules. However, the diffusion of larger molecules such as proteins is limited by their molecular sizes and loads. Electron microscopy and gel chromatography showed that the pore size of alginate gels is in the range of 5–200 nm. The type of gel formation mechanism (external or internal) affects the size of the gel pores. The pore size is also determined by the composition of the alginate monomers. The porosity of the gel increased with a high content of G monomer because the gel with high G monomer adopts a more open pore structure that is less sensitive to shrinkage [35,60].

### 4.5. Release Characteristics

In general, low-molecular-weight solvent-soluble substances, such as drugs, vitamins, and sugars that are smaller than the pore size of alginate gels can diffuse freely into the gel particles. When the alginate gel matrix disintegrates, the base material is released by erosion. Gel decomposition occurs at high pH or in the presence of cationic chelating compounds such as EDTA and citrate. Under these conditions, the alginate matrix swells due to the ion exchange of the gel cation ion with the Na ions present in the environment. Due to the swelling of the gel, the core material is released due to the reduction of crosslinking. By eroding the gel matrix, diffusion occurs more rapidly. One of the factors that affect the release of compounds from alginate gel particles is the type of cation. For example, in alginate gels formed with Ba^2+^ or Al^3+^, smaller pores are observed compared to Ca^2+^ alginate gels, and smaller pores release water-soluble compounds. Smaller pores cause the release of water-soluble compounds to be delayed. Another effective factor in the diffusion properties of alginate gels is the method of gel formation. The homogeneous structure of the gel formed by the internal method, due to the uniform pore size throughout the structure, has a faster rate of diffusion of compounds from the gel, but the gel with the heterogeneous structure made by the external gelation method with higher cross-bond density on the external surface delays the release of the main materials. Crosslink density in alginate gels is also very important for the release of bioactive substances. The permeability of the gel matrix depends on the concentration of the cations that make up the gel. Usually the permeability of the alginate gel decreases by increasing the gelling Ca^+2^ concentration [35].

### 4.6. Syneresis and Swelling

During alginate gel formation, water attached to the internal gel structure by hydrogen bonds is trapped in the gel matrix. When an external force contracts the gel, water leaves the gel matrix and syneresis occurs. In food hydrocolloids, hard and brittle gels are more prone to syneresis than elastic gels. Higher syneresis was observed in alginate gels with a higher proportion of alternating GM blocks compared with those with high M block contents. It has also been found that low molecular weight alginates form a rigid gel structure that resists the forces of deformation (contraction), leading to syneresis. Therefore, gels made with high molecular weight alginate had higher syneresis than those formed with low molecular weight alginates [61].

In a system where the alginate gel is saturated with Ca^2+^ ions, it appears that the syneresis is negligible. The rate of swelling of alginate gels varies under different conditions and is influenced by different parameters. One of these factors is the amount of calcium ions. Studies have shown that the swelling capacity of alginate beads decreases with increasing Ca^2+^ concentration. The type of cation is also important and Ba^2+^ ions-induced gels showed significant swelling compared to Ca^2+^ ion-derived gels. The mean diameters of microspheres of low viscosity high-guluronic acid alginate cross-linked with Ba^2+^ and Ca^2+^ were 14.1 and 38.1%, respectively. This difference was attributed to the higher affinity of Ba^+2^ for G blocks. The swelling of alginate beads decreases with high amounts of G blocks. A study has shown that the mean change in the diameters of high-mannuronic acid alginate microspheres was 30% more those made with high-guluronic acid alginates [35,62].

The size of the alginate gel particles also depends on the pH. It decreases at low pH and swells above pH 6.6. The amount of swelling also depends on the amount of alginate blocks. The increase in size of calcium–alginate changes (due to swelling) for calcium–alginate pellets were in the order of 0.24, 2.6 and 2.97 times of their original size at pH 1.5, 4 and 6.6 [63].

### 4.7. Effects of pH

pH plays a critical role in various processes like preparation and formation of hydrogels, swelling, release, and degradation. Sensitivity to ambient pH is due to the presence of -COOH groups in the alginate polymer structure [64]. The initial pH value of the native alginate solution is usually close to 7.0, and the flow index decreases with a decrease in the pH. The solution at a pH equal to 7.0 has a Newtonian, an intermediary behavior between Newtonian and non-Newtonian shear thinning at pH 6.0 and 5.0, and a clearly shear thinning behavior at lower pH (4.5 and 3.0). Below a pH value of 3.0, alginic acid precipitated [3]. The behavior of alginate at different pHs is expressed in such a way that at pH less than pKa (is Equal 3.4), an insoluble structure occurs because the COOH-acid groups are non-ionized. At pH above 4.4, the –COOH group ionizes, therefore, the negative charge increases the electrostatic repulsion leading to the expansion of the polymer chain and the swelling of the hydrophilic matrix [64]. Alginate gel particles undergo morphological and chemical changes at different pHs. At low pH, the gel particles shrink and the pore size of the gel decreases, but at a pH above neutral, it is the opposite. The pore size increases and the gel particles swell. Alginate gel dissolves after prolonged exposure to high pH. The results indicate that when the pH was reduced from 4 to 1, a decrease in the particle size of the alginate gel was observed. The mechanism by which low pH causes the gel to shrink is unclear. However, low pH suppresses the separation of carboxyl groups in macromolecules. Carboxyl groups that are protons form a smaller gel network due to the reduced electrostatic repulsion between alginate polymers [35]. The intrinsic viscosity of alginates varied from 8.6 to 15.2 (dL/g) [18].

### 4.8. Rheology of Alginate Solutions

The flow behavior, thixotropy and dynamical viscoelastic properties are the most important rheological characteristics of polysaccharides. Contrasting behaviors (Newtonian or non-Newtonian) have been observed at the same concentrations of alginate solutions in water. While the behavior of sodium alginate solutions (with concentrations between 1.0 to 5.0% *w*/*v*) of *Nizimuddinia zanardini* was reported as Newtonian or very low shear thinning, a pseudo plastic behavior was observed above a critical shear rate in G-rich commercial sodium alginate solutions (between 1.0 to 3.0%, *w*/*v*) [49,65]. The viscosity of solutions is highly dependent on temperature, molecular conformation of the polymer, the ionic strength of the solvent and the amount of NaCl in solutions. Alginate solutions exhibit higher viscosities at low temperatures, low ionic strengths and high NaCl concentrations. Inversely, alginate solutions exhibit lower viscosities at high temperatures and higher ionic strength due to the increased intermolecular distances as a result of thermal expansion and a more compacted conformation, respectively. Moreover, the addition of NaCl to sodium alginate solutions significantly increases their viscosity (due to the inter-chain associations) [65,66,67,68].

The storage modulus (G’) and the loss modulus (G”) that can be evaluated by oscillatory analysis are sensitive to molecular structure and interactions in solutions and are frequency and temperature dependent. The increasing of temperatures from 5 to 35 °C reduced the G’ and G’’ of sodium alginate solutions (2.5% (*w*/*v*)); upside down these two parameters increased with the increase of angular frequency under small deformation conditions. Loss modulus values were always slightly larger than the storage modulus ones at low frequency and tended to approach each other at high frequency. The behavior of aqueous sodium alginate solutions was predominantly more viscous than elastic and showed a fluid-like viscoelastic behavior [8,65].

## 5. Applications

Various grades of seaweed alginates are currently on the market and are classified depending on their distribution pattern of M- and G-blocks, molecular weight, purity and composition [8]. The Food Standards Agency in 2002 gave E numbers to alginates as food additives that have been approved for use throughout the European Union (EU), including alginic acid, sodium, potassium, ammonium and calcium salts of alginate, which are E400–E404, respectively [69]. Alginic acid esters and propylene glycol alginates (PGAs) are known as E405. They are used in the food industry depending on their varying degrees of esterification and viscosity at lower concentrations that those of traditional alginates [69,70]. The alginates extracted from seaweed is usually sodium alginate. The United States began large-scale industrial production of sodium alginate in 1929 and then in 1983, the Food and Drug Administration of the United States (USFDA) approved the direct use of sodium alginate as a food ingredient [1]. It is used in the biotechnology industry as thickener and a gelling agent but also as a colloidal stabilizer. Alginate has also the unique capacity to be used as a matrix to trap or deliver a variety of molecules or particles [53]. Sodium alginates are now widely used in the medical, cosmetic, textile, pharmaceutical (Table 3), and food industries due to their rheological properties. Industrial applications of alginates are related to their ability to retain water as well as their gel forming, viscosity, and stabilizing properties. Biotechnological applications are based on instantaneous physical bonding that is almost independent of temperature and sol/gel transfer in the presence of polyvalent cations (such as Ca^2+^) in aqueous medium. It is a simple and cost-effective process that results in the preparation of a gel with highly adjustable mechanical properties and its capacity to store large amounts of fluids, which is suitable as a stationary matrix for various applications such as drug delivery, genes or cells for tissue engineering and applications, treatment and resuscitation and delivery to a specific site of mucosal tissue due to the adhesion of alginates [2]. The use of hydrocolloid gel particles is potentially useful in the food, chemical and pharmaceutical industries. Alginate gel particles are one of the most common hydrocolloid gels that are produced due to their biocompatibility, non-toxicity, biodegradability, low cost and ease of use. They are also of great value for their use in encapsulation.

Encapsulation with alginate gel particles gives protective benefits to cells, DNA, nutrients and microbes. The slow release of flavors, minerals and drugs can also be achieved by encapsulating alginate gel. Based on the results of research, it has been determined that the technological properties of extracted alginates with higher viscosity are more suitable for the production of resistant gels in food and cosmetics [11,18].

Other uses for alginates include their utility as a low-cost protein source, ferrogels for intelligent transmission for cancer treatment, alginate-calcium thin films for refining heavy metal ions, and biomaterials for tissue regeneration [12].

### 5.1. Food Applications

Alginate is commonly used in the food industry to modify some food characteristics such as rheology (thickening), water binding capacity, stabilizing emulsion and film formation [35]. Combined active compound and alginate coating or thin-layered structures are used to increase the storage period of tomato (*Solanum lycopersicum* L.) [77], mushrooms (*Agaricus bisporus*) [78], shrimp [79], turkey fillet [80], chicken thigh meat [81], low fat cut cheese [82] and meat [83]. Because of thickening and gelling properties, it can be used in sauces, jam, marmalade, syrups, ice cream toppings and in fruit pies, and animal food. In the production process of ice cream, the use of propylene glycol alginate in low concentrations cause to soft tissue, low ice crystals and gives desirable feeling to customers during production to consumption. Another alginate application is stabilizing fruit drinks and beer. Alginate is useful in mayonnaise and salad dressing which we know of as water-in-oil emulsions [84]. Calcium alginate structures are considered by the meat industry as an alternative to natural casings from animals. In a 2015 study on the replacement of alginate structures with natural coatings in fermented sausages, at 12 °C it was found that alginate coatings could be a suitable alternative to natural coatings [85]. The physiological and rheological properties of alginates, as well as their applications as stabilizers, thickeners, gels or pharmaceutical additives, are strongly influenced by the composition of uronic acids (M/G ratio) and the distribution of monomers along the chains [26]. Alginate is used due to its low water solubility and high viscosity, especially in food products. This polysaccharide has antioxidant properties and prevents the unpleasant role of free radicals and oxidative damage in foods and improves the quality of nutrition. Its structural properties such as molecular weight, monosaccharide composition and glycosidic branching affect its antioxidant activity. The molecular weight and M/G ratio of alginates play an important role in their ability to inhibit free radicals. Low molecular weight polysaccharides were hypothesized to have more reducing hydroxyl groups (by mass) to accept and scavenge free radicals. On the other hand, the higher proportion of G monomers increases the antioxidant activity because the diaxial bonding in these blocks may cause a hindered rotation around the glycosidic bond. As a result, the flexibility of G-blocks increases, thereby affecting the availability of hydroxyl groups in sodium alginate and the ability to donate H-atoms. Alginates also have the ability to inhibit lipid peroxidation of phosphatidylcholine and linoleate liposomes, protect NT_2_ neurons from H_2_O_2_-induced neurotoxicity, and inhibit free radical chain reactions [3]. A study on sodium alginate from the Tunisian seaweed *Gongolaria barbata* (formly *Cystoseira barbata*) in 2015 found that it was composed of 37% manuronic and 63% guluronic acids. It is less sensitive to temperature changes and is more stable at an acidic pH. The compound has also been studied for its antioxidant properties and has moderate antioxidant activity and strong protective activity against DNA breakage. Therefore, this alginate could be used as a natural substance in the food or pharmaceutical industries [37]. Alginate is very useful to encapsulate some strains of live cell of probiotics in both intestinal tract and food products [86]. The microencapsulation technique protects live bacteria during storage time [87]. Generally, alginate can be used as an additive (thickener, emulsifier, stabilizer, etc.) at very low concentrations in milk chocolate and as an ingredient in functional foods (probiotics and prebiotics) [86].

### 5.2. Non-Food Applications

The ability to form alginate gels in the presence of polyvalent cations, biocompatibility and biodegradation has made this polymer a very special material for medical applications. Alginate microparticles are a potential biological material for improving the quality of life of inflammatory bowel disease patients due to their remarkable cross-linking and adhesion capabilities, which is a good choice for the colon delivery system. Other properties of alginate include its use with other polymers as well as microcoating techniques [88]. Three-dimensional and four-dimensional printings, also known as bioprinting, are performed using alginate hydrogels derived from brown algae. It is used in the engineering of body tissues, for example bones, cartilage (joints), brain (nerve), ear, heart, eyes (cornea), and give access to natural organs to study various types of diseases [89]. Low molecular weight alginates have been shown to be effective in preventing obesity, hypercholesterolemia and diabetes [15]. In a study, it was found that oxidized alginate-based hydrogels (OA) are used for tissue engineering applications including bone, cartilage, blood vessels, corneas and other soft tissues. Oxidation of alginate leads to cleavage of C-C bonds and the formation of aldehyde groups in oxidized monomer units. The molecular weight of OA is lower than that of pure alginate due to the degradation that occurs during the oxidation process. The biodegradation of OA is increased because the aldehyde groups formed are sensitive to hydrolysis. OA has fewer mechanical properties than pure alginate, and the aldehyde groups formed may be slightly toxic to cells. However, the aldehyde groups in the polymer chain allow covalent cross-linking with other materials through a Schiff-based reaction with amine. Covalent cross-linking of OA leads to a reduction in the number of free aldehyde groups that increases the mechanical properties of the gel and improves the biocompatibility of OA. Therefore, OA-based hydrogels can be prepared with suitable properties for various medical applications through the regulation of the composition of the ingredients in the gel or a change in the degree of oxidation [90]. Injectable alginate hydrogels and their composites can be used to regenerate bone tissue [91]. In 2015, scientists were able to produce artificial bone using tissue engineering sciences. Bone is a complex tissue of which nanohydroxyapatite and collagen are its major proteins. Alginate is an anionic polymer that is important due to its biocompatibility and gel formation properties and is used in biomedical science, especially in bone tissue engineering. Several composites such as alginate-polymer (PLGA, PEG and chitosan), alginate-protein (collagen and gelatin), alginate-ceramic, alginate-bioglass, alginate-biosilica and RGD peptide compounds have been studied to date. These alginate composites have shown good properties in terms of porosity, mechanical strength, cell adhesion, biological compatibility, cell proliferation, increased alkaline phosphatase, increased minerals and bone differentiation. Hence, alginate-based composite biomaterials will enhance bone tissue regeneration [92]. From a study conducted in 2015, it was concluded that alginate can be used in fuel cells as a substitute for polyelectrolyte membranes, especially in low-to-medium temperature polymer electrolyte fuel cells, direct methanol fuel cells, polymer-alkali electrolyte fuel cells and biofuel cells. Alginate compounds are hydrophilic and can be easily modified to create the required surface properties, such as low methanol permeability and proton conductivity (such as Nafion membrane samples). These biopolymers play an important role in polyelectrolyte (PEC) structures and have always performed better with all modifications. In addition, the production of alginate is relatively inexpensive [92]. Among the various polymers, alginate has found many applications in food, medicine and packaging. Packaging plays an essential role in various industries. For example, in drug delivery systems, mesh made of nanofibers produced by electrospinning is very desirable and has received much attention. Electrospinning in medical science for the production of non-woven structures at the nanoscale is based on the use of biopolymers and natural materials with a combination of drugs (such as naproxen, sulfixoxazole) and essential oils with antibacterial properties (such as tocopherol, eugenol). This is an interesting method that, due to its ability to produce nanobase-scale materials and structures of exceptional quality, allows the materials to be encapsulated and the drugs and biologically active substances to be placed on the polymer nanofibers. Therefore, among the various materials studied, alginates have a very high potential in electrospinning [93]. Pollution of aquatic environments with heavy and toxic metals entering the environment from mines and industrial effluents is a major cause for concern. Treatment of these polluted effluents is also a priority [20]. Achieving environmental goals requires innovative technologies in water and wastewater treatments. Adsorption technology is considered as one of the most effective and environmentally friendly methods to eliminate pollutants that are difficult to destroy in the environment. Alginate-based composites (combining alginate gels and other polymers, natural and engineered nanoparticles, and microorganisms), as a low cost and highly efficient adsorbent, are widely used to remove heavy metals, industrial paints, pesticides, antibiotics and other contaminants in water and wastewater [94]. The use of natural biomass as a substrate for chelating metal ions is called biosorption and refers to a passive process, or rather, a non-metabolic mediator. *Sargassum* brown algae has been shown to have the required mechanical properties, chemical affinity and adsorption capacity for bonding metals such as Cd, Au, Cu, Fe, Ni, Pb and Zn in an efficient, reversible and cost-effective manner from polluted waters [11,20,32]. The biosorption capacity of these algae for heavy metals is mainly due to the presence of alginate in them. In this process, both the content and the composition of the alginate, the amounts of guluronic and mannuronic acids and the different block contents in the calcium alginate network are very important. The presence of G monomer in pure alginates and in brown algae is very important because it leads to good separation of divalent ions [34]. The use of hydrogel alginates has expanded its applications in the food industry, wastewater treatment, and as an adsorbent to remove heavy metals from contaminated water [10]. Highly adsorbable alginate-based hydrogels with mechanical stability and viscoelastic properties are used as wound dressings [95]. Sodium alginate is used in the textile and paper industries as a thickener, stabilizer and gel [9].

### 5.3. Pharmaceutical Applications

Recent advances have shown that alginates can be used as a matrix for three-dimensional tissue cultures, adjuvants for antibiotics, cell transplantation in diabetes, or alginate-based drugs in the treatment of neurological diseases, as well as in antimicrobial and antiviral therapy [95]. Alginate-based particles are used as one of the most important factors in drug delivery due to their inherent properties such as good biocompatibility and biodegradability. In addition, their low cost, availability, natural resource, flexibility and cell gel transfer properties make alginate one of the ideal materials for the production of particles and nanoparticles with different applications [96]. Alginates can also be used in carriers for drug delivery. In fact, nanotechnology enables drug delivery at the nanoscale and even minimizes side effects. The main purpose of using nanotechnology for drug delivery is mainly to increase drug loading and reduce the toxicity of pollutants and to maintain high safety and increase therapeutic effects and biocompatibility [97]. Pharmacological studies have shown that polysaccharides extracted from *Sargassum fusiforme* significantly reduced the content of total cholesterol, triglycerides, and low-density hypoprotein cholesterol in mice [98]. The crude extract of *Sargassum polycystum* has been shown to have a significant effect on the prevention of severe fat disturbances and the metabolism of acetaminophen-stimulated enzymes during liver injury [99]. Alginate is used as a matrix to encapsulate or release cells and drugs. Its microparticle form is also used for antigen release systems because it is non-toxic and, most importantly, FDA-approved. *Sargassum vulgare* extract has shown an inhibitory activity of syncytium against human T-cell lymphotropic virus (HTLV-1), and may be useful in preventing infection [32]. Alginates also have anticoagulant activity, which makes them effective in biomedicine [15]. Alginate composites can be used for in vivo delivery of cells and proteins which improves tissue, as anti-glycemic supplements in diets for their antioxidant and antibacterial activities and as control protein delivery agents due to their porosity and gelling ability [14,100,101,102].

## 6. Conclusions

These days, seaweed has received a lot of attention due to the wide range of valuable products in it such as polysaccharides and oligosaccharides. Alginate polysaccharides and oligosaccharides have valuable properties such as biocompatibility, nontoxicity, biodegradability and functional versatility with various matrices and substrates. They are used in food, agriculture and the pharmaceutic industry and as a growth stimulant for plants. The properties of alginates prove that they are efficient candidates to be used in various applications ranging from additives to food and beverages up to scientific applications. Apart from the food and medicinal industries, the use of alginates has to be explored in many other fields. For example, in recent years the results of clinical trials on heart patients showed that alginate appears to improve patient quality of life. Although polysaccharides from marine macroalgae have comprehensively reported potential in valuable biomedical applications, commercial products are scarce on the market because there are still challenges with the development of these biomaterials, including the transfer of technology, production process expansion, regulatory safety requirements, environmental concerns and consumer acceptance. Despite its easy production, it must be produced by adaptable green scale production systems on an industrial scale in high purity and directly from algal biomass.

## Figures and Tables

**Table 1:** The yield (% dry weight) and M/G ratio of alginates extracted from various species of brown seaweeds.

Seaweed Species	Place of Collection	Extraction Yield	M/G Ratio	References
*Sargassum asperifolium*	Egyptian Red Sea coast	12.0	0.69	[18]
*Sargassum dentifolium*	Egyptian Red Sea coast	3.3	0.52	[18]
*Sargassum duplicatum*	Saudi Arabia	32.5	0.86	[19]
*Sargassum fluitans*	Sargasso Sea of the north-west Atlantic Ocean	21.1–24.5	0.52–0.57	[20]
*Sargassum hemiphyllum*	Guangdong Province	23.0	1.06	[17]
*Sargassum henslowianum*	Guangdong Province	17.8	0.82	[17]
*Sargassum horneri*	Dalian	11.5	0.64	[17]
*Sargassum ilicifolium*	Egyptian Red Sea coast	4.3–17.2	0.25–0.82	[18]
*Sargassum mangarevense*	Tahiti, French Polynesia	9.3 ± 1.7	1.42 ± 0.24	[21]
*Turbinaria ornata*	Tahiti, French Polynesia	19.2 ± 1.3	1.25 ± 0.20	[21]
*Turbinaria ornata*	Hainan Island	20.6	0.89	[17]
*Sargassum mcclurei*	Guangdong Province	23.6	1.4	[17]
*Sargassum microphyllum*	Taiwan	20.3–23.5	-	[22]
*Sargassum miyabei*	Qingdao	10.5–18.1	0.62–1.10	[17]
*Sargassum oligocystum*	Australia	16.3–20.5	0.49–0.62	[20]
*Sargassum pallidum*	Qingdao	10.4	1.26	[17]
*Sargassum patens*	Guangdong Province	16.0	1.59	[17]
*Sargassum polycystum*	South India	17.1–27.6	0.56–0.74	[23]
*Sargassum siliquastrum*	Guangdong Province	18.1	1.13	[17]
*Sargassum thunbergii*	Qingdao	12.8	0.78	[17]
*Sargassum vulgare*	Saudi Arabia	30.2	0.71	[19]
*Turbinaria murrayana*	Saudi Arabia	40.1	1.09	[19]
*Sargassum wightii*	India	21.1–33.1	-	[24]
*Sargassum natans*	Ghana	23 ± 1.6	0.6	[25]
*Sargassum vulgare*	Ghana	17 ± 4.4	0.6 ± 0.1	[25]
*Padina gymnospora*	Ghana	16 ± 0.7	1.5 ± 0.2	[25]
*Padina antillarum*	Ghana	22 ± 1.1	1.5 ± 0.1	[25]
*Zonaria sp.*	Madagascar	10.2–30.0	0.41	[26]
*Chnoospora sp.*	Madagascar	9.2- 50.8	0.51	[26]
*Spatoglossum sp.*	Madagascar	9.7–17.4	0.75	[26]
*Spatoglossum sp.*	Madagascar	22.0–30.5	0.68–1.09	[26]
*Cystoseira compressa*	Tunisia	21.65 ± 1.5	0.77	[27]
*Cystoseira humilis*	Morocco	5.43–19.21	1.46	[28]
*Fucus vesiculosus*	Quebec	16.2 ± 3.2	1.17	[29]
*Ascophyllum nodosum*	Quebec	24.0 ± 0.3	0.61	[29]
*Saccharina longicruris*	Quebec	20.0 ± 1.1	0.79	[29]
*Laminaria digitata*	Morocco	35.2 − 51.8	1.12	[30]
*Macrocystis pyrifera*	Argentina	33	1.17	[4]
*Laminaria digitata*	France Atlantic ocean	-	1.5	[31]
*Sargassum vulgare*	Brazil	-	1.27	[32]
*Sargassum turbinarioides Grunow*	Madagascar	10	0.94	[33]
*Sargassum thunbergii*	Korea	-	0.53	[34]

**Table 2:** Uronic acid sequences (blocks) of alginates.

Source	^1^ F_G_	^2^ F_M_	^3^ M/G	^4^ F_GG_	^5^ F_MM_	^6^ F_MG_	^6^ F_GM_	References
*S. natans*	0.67	0.33	0.49	0.59	0.24	0.09	0.09	[12]
*C. *schiffneri**	0.91	0.09	0.10	0.88	0.06	0.03	0.03	[37]
*C. compressa*	0.56	0.44	0.77	0.53	0.40	0.03	0.03	[27]
*N. zanardini*	0.47	0.53	1.11	0.44	0.47	0.06	0.06	[3]
*L. digitata*	0.47	0.53	1.12	0.41	0.47	0.06	0.06	[30]
*C. humilis*	0.41	0.59	1.46	0.21	0.40	0.19	0.19	[28]
*F. vesiculosus*	0.46	0.54	1.17	0.26	0.33	0.21	0.21	[29]
*S. longicruris*	0.56	0.44	0.79	0.24	0.11	0.33	0.33	[29]
*A. nodosum*	0.62	0.38	0.61	0.31	0.08	0.30	0.30	[29]
*S. vulgare*	0.44	0.56	1.27	0.43	0.55	0.02	0.02	[32]
*S. fluitans*	0.46	0.54	1.18	0.28	0.36	0.36	0.36	[20]
*S. oligocystum*	0.62	0.38	0.62	0.55	0.31	0.14	0.14	[20]
*C. myrica*	0.69	0.31	0.45	0.59	0.21	0.10	0.10	[18]
*C. trinode*	0.63	0.37	0.59	0.50	0.24	0.13	0.13	[18]
*S. latifolium*	0.55	0.45	0.82	0.51	0.41	0.04	0.04	[18]
*S. polycystum*	0.82	0.18	0.21	0.77	0.12	0.10	0.10	[34]
*S. filipendula*	0.84	0.16	0.19	0.76	0.07	0.16	0.16	[33]
*L. japonica*	0.35	0.65	1.86	0.21	0.51	0.14	0.14	[38]

^1^ F_G_ = G/(M + G), ^2^ F_M_ = M/(M + G), ^3^ M/G = (1-F_G_)/F_G_, ^4^ F_GG_ = GG/(M + G), ^5^ F_MM_ = MM/(M + G), and ^6^ F_GM_ = F_MG_ = MGG/(M + G).

**Table 3:** Some examples of pharmaceutical products based on alginates.

Product	Company	Route of Administration	Main Ingredients	Description	Indications	References
Purilon Gel^®^ gel	Coloplast	Dermal	Calcium alginate and sodium carboxymethylcellulose	Provides moist environment at wound surface	Indicated in conjunction with a secondary dressing for necrotic and sloughy wounds and first and second degree burns	[71]
Algivon^®^ dressing	Advancis Medical	Dermal	Calcium alginate dressing impregnated with Manuka honey	Binds of exudate, regeneration	Sloughy, necrotic, and malodorous wounds	[72]
Gaviscon^®^ Double Action Liquid	Reckitt Benckiser Healthcare Hull, UK	Oral	0.25 g sodium alginate, 162.5 mg calcium carbonate, and 106.5 mg sodium bicarbonate per 5 mL	Creates a mechanical barrier between the stomach and the esophagus; regenerates mucous membranes of the esophagus and ensures its protection; accelerates gastric movement	Adult reflux treatment	[73]
Progenix putty^®^	Medtronic Spinal & Biologics	Periodontal	Demineralised bone matrix in type-1 bovine collagen and sodium alginate	Regeneration, complementation of bone losses; periodontal diseases	Gaps or bony voids of the skeletal system	[74]
Natalsid^®^ suppositories	STADA	Rectal	Sodium alginate	Anti-inflammatory local action	Chronic haemorrhoids, proctosigmoiditis, and chronic anal fissures after surgical interventions in the area of the rectum	[75]
ChondroArt 3D^®^ injection	Arkopharma	Arthroscopic	Autologous chondrocytes situated on a hydrogel scaffold built from connection of alginate and agarose	Increase production and growth of cartilage	Degenerative diseases of joints and backbones (osteochondrosis, osteoarthrosis)	[76]

## Data Availability

Not applicable.
